# Understanding the Specification Mechanism of the Megaspore Mother Cell in *Arabidopsis*

**DOI:** 10.3390/biology15171507

**Published:** 2026-09-03

**Authors:** Lian Yu, Simin Ma, Xinpeng Xi, Peiyang He, Hanyang Cai

**Affiliations:** Fujian Provincial Key Laboratory of Haixia Applied Plant Systems Biology, State Key Laboratory of Agricultural and Forestry Biosecurity, Haixia Institute of Science and Technology, College of Life Sciences, Agriculture and Forestry University, No. 15 Shangxiadian Road, Cangshan District, Fuzhou 350002, China; yulian20011108@163.com (L.Y.); msm18159875525@163.com (S.M.); 18235795238@163.com (X.X.); 15090921733@163.com (P.H.)

**Keywords:** female gametophyte, cell specification, fate determination, signal transduction, non-cell-autonomous regulation, lateral inhibition

## Abstract

The megaspore mother cell (MMC) is the founding cell of the plant female germline, and its specification is a critical step in sexual reproduction of flowering plants. Recent advances in single-cell transcriptomics, live imaging, and gene editing have significantly advanced our understanding of the molecular mechanisms underlying MMC specification in *Arabidopsis thaliana*. Here, we systematically review the latest findings from three integrated perspectives. Cell-autonomous regulatory mechanisms that establish MMC fate; non-cell-autonomous signals originating from somatic cells that position and permit MMC specification; and mechanisms by which neighboring somatic cells maintain their own fate and restrict MMC number. We also outline key open questions and offer perspectives on future research directions. This review provides a comprehensive reference for understanding the molecular-level mechanisms governing female germ cell fate determination in plants.

## Simple Summary

Flowering plants produce seeds after successful sexual reproduction. Each plant ovule normally forms only one female reproductive precursor cell, which further develops into the seed-forming structure. When this developmental control fails, extra reproductive-like cells appear and seed production is disrupted. This review summarizes how plant cells precisely select one single cell to become the female reproductive precursor. We describe internal genetic and chemical signals within the precursor cell, and messages sent from surrounding tissue cells to restrict extra cells from gaining reproductive identity. Multiple layers of genetic and chemical safeguards work together to maintain correct cell fate. We also list unanswered questions in this field and suggest promising future research directions. Understanding how plants establish their female reproductive cells can help improve seed yield for crop plants and support agricultural production.

## 1. Introduction

**Figure 1:**
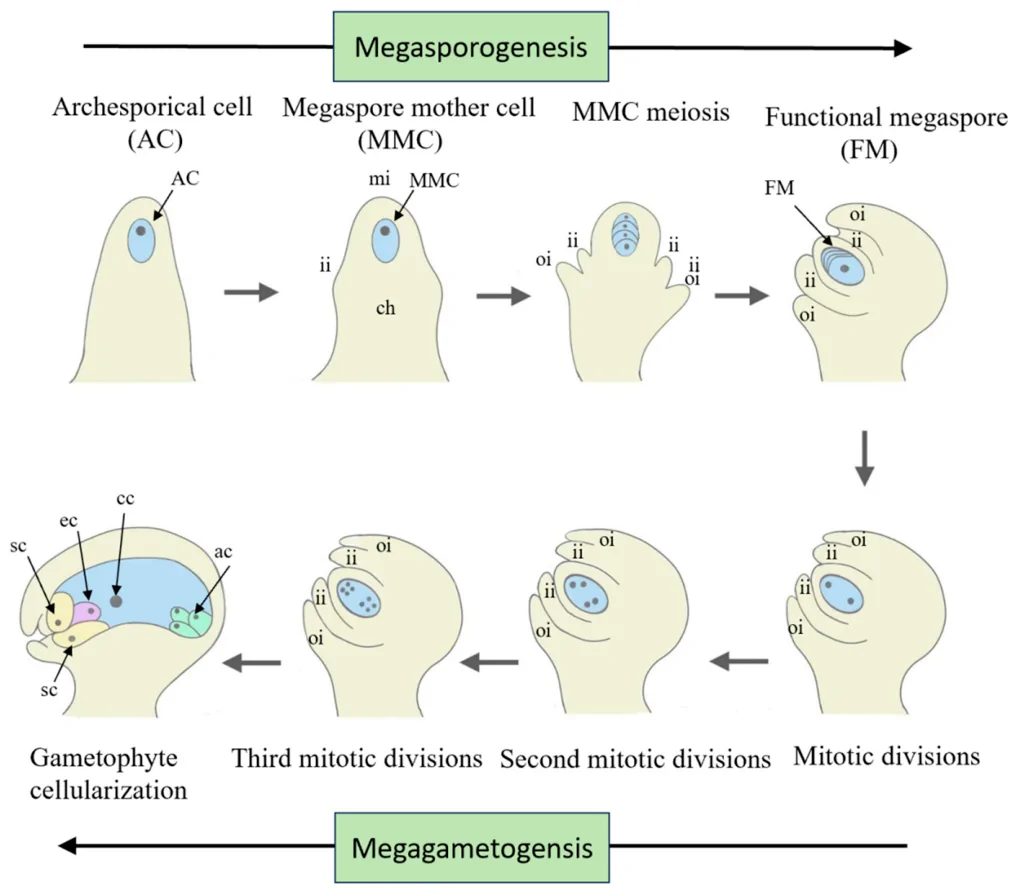
Female germline development in *Arabidopsis* ovules is divided into two sequential phases: megasporogenesis and megagametogenesis. Megasporogenesis starts with the specification of an archesporial cell (AC) from the subepidermal nucellar tissue of the ovule primordium, which differentiates into a megaspore mother cell (MMC). The MMC executes meiosis to produce four haploid megaspores; only the chalazal-located megaspore survives and develops into a functional megaspore (FM), whereas the three micropylar megaspores degenerate completely. The subsequent megagametogenesis stage consists of three successive mitotic divisions of the FM, followed by cellularization to generate the mature seven-celled, eight-nucleate female gametophyte (embryo sac). Abbreviations: mi, micropyle; ch, chalaza; oi, outer integument; ii, inner integument; sc, synergid cell; ec, egg cell; cc, central cell; ac, antipodal cell. *Note: This is a simplified conceptual schematic; relative sizes of cells and ovule-related structures are not drawn to scale*.

Reproductive development in flowering plants is a complex and exquisitely regulated process involving the alternation between diploid (sporophytic) and haploid (gametophytic) generations [1]. Within this process, the MMC represents the first specified cell of the female germline and carries the critical mission of transitioning from a somatic to a germline identity [2]. The MMC originates from a single subepidermal L2-layer cell in the distal region of the nucellus of the ovule primordium. This cell elongates and expands to form the archesporial cell (AC), which subsequently differentiates into the MMC. This remarkable cell-size expansion also generates mechanical tension that may trigger mechanosensing-related interactions with adjacent nucellar somatic cells, providing potential physical cues for MMC specification. Ultimately, the MMC undergoes meiosis to produce four megaspores, of which only one functional megaspore (FM) survives and develops into the female gametophyte (embryo sac), thereby establishing the foundation for fertilization and seed formation [3,4] (Figure 1).

MMC specification is governed by a multi-level, multi-dimensional regulatory network. The establishment of the “one ovule, one megaspore mother cell” principle relies on the coordinated action of cell-autonomous programs that promote MMC fate and non-cell-autonomous signals from surrounding cells that both permit and restrict this fate [1,5]; The geometry, cell division patterns, and growth dynamics of the ovule primordium provide a physical framework for MMC specification. High-resolution three-dimensional morphometric analyses have revealed that MMC enlargement is closely associated with its position within the nucellar primordium, and differential expansion of apical and basal cell walls imparts a distinctive trapezoidal shape to the cell [6,7]. Epigenetic modifications—including DNA methylation, histone variant deposition, and small RNA-mediated gene silencing—play crucial roles in both maintaining and restricting MMC fate [8,9].

**Figure 2:**
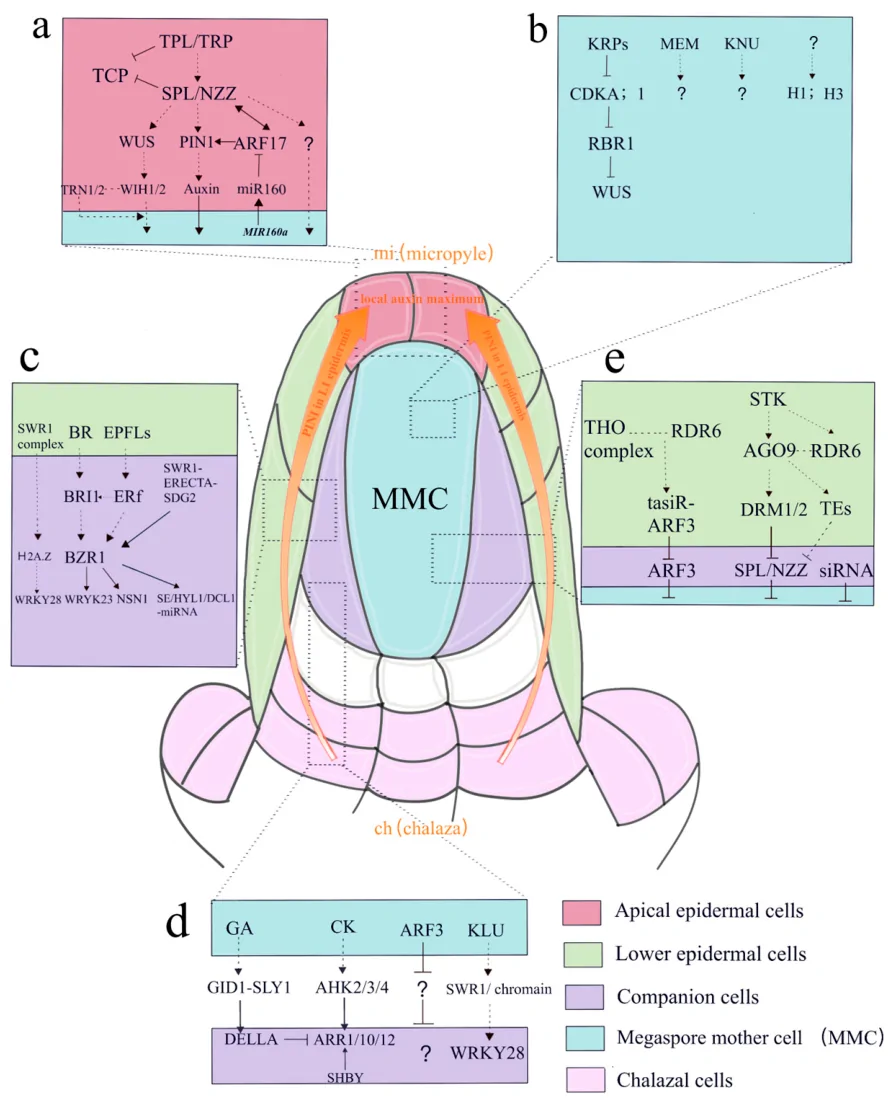
Hormonal-signaling regulatory networks operating in distinct cell-types during Arabidopsis ovule primordium development. (**a**) In apical epidermal cells, the TCP-TPL/TRP complex represses SPL/NZZ, which modulates the PIN1-ARF17-miR160 auxin feedback loop to maintain WUS expression. (**b**) In the megaspore mother cell (MMC), KRPs inhibit CDKA;1 activity, and the RBR1 pathway represses WUS; MEM, KNU, and histone variants H1/H3 are also implicated in this regulatory cascade. (**c**) In companion cells, the SWR1 complex mediates H2A.Z deposition; BR signaling transduced via BRI1-BZR1 coordinates with ERF and the SWR1-ERECTA-SDG2 module to regulate downstream targets. (**d**) In chalazal-derived tissues, GA promotes DELLA degradation through GID1-SLY1; CK activates ARR1/10/12 via AHK2/3/4, whose activity is repressed by SHBY; KLU modulates WRKY28 via chromatin-remodeling events, and ARF3 integrates auxin with GA/CK hormonal inputs. (**e**) In lower epidermal cells, the THO-RDR6 complex generates tasiR-ARF3 to repress ARF3 in companion cells, whereas STK-AGO9-RDR6 mediates RdDM to silence DRM1/2 and transposable elements. PIN1-dependent auxin transport (orange arrows) establishes a local auxin maximum at the micropylar end. Solid arrows indicate activation; flat-ended lines represent inhibition; dashed arrows denote indirect or putative regulatory interactions. Auxin is synthesized in apical epidermal cells; BR is produced in epidermal cells and perceived in companion cells; GA and CK originate mainly from chalazal maternal tissues. The question mark (?) indicates molecular links that remain uncharacterized and require further experimental vali-dation. *Note: This figure was adapted from Cai et al. (2022) under the CC BY 4.0 license (Positional signals establishment in the regulation of female germline specification, Seed Biology, https://doi.org/10.48130/SeedBio-2022-0006*).

In recent years, the rapid development of single-cell RNA sequencing (scRNA-seq), high-resolution three-dimensional morphometric analyses, and gene editing technologies has enabled scientists to dissect the molecular events of MMC specification at single-cell resolution [10,11]. These studies have uncovered, at the spatial molecular level, the transcriptional regulatory networks underlying MMC specification and elucidated the coordinated mechanisms of cell–cell communication, hormonal signal integration, and epigenetic reprogramming during female germline establishment. In this review, we systematically summarize recent advances in understanding MMC specification mechanisms in *Arabidopsis*, focusing on three integrated levels: cell-autonomous regulatory mechanisms, non-cell-autonomous signals from somatic cells to the MMC, and the mechanisms by which neighboring somatic cells maintain their own fate and restrict MMC number (Figure 2). We also offer perspectives on future research directions in this field.

While *Arabidopsis* serves as the primary model for dissecting MMC specification, emerging evidence suggests that core regulatory modules are partially conserved across angiosperms. In rice (*Oryza sativa*), MSP1 (*MULTIPLE SPOROCYTE 1*), a homolog of the TPD1/EMS1 signaling module, restricts the number of sporogenous cells in both anthers and ovules, and its loss-of-function leads to multiple MMC-like cells [12]. Similarly, in maize (*Zea mays*), the MAC1 (MULTIPLE ARCHESPORIAL CELLS 1) gene exerts a conserved restrictive function in germline development. However, the extent to which the *SPL/NZZ-WUS-KRP-RBR1* axis and the *ERf-EPFL-BZR1* module are functionally conserved in crops remains largely unexplored. Future comparative studies in rice, maize, and tomato will be essential to determine whether the regulatory principles established in *Arabidopsis* represent universal mechanisms or lineage-specific adaptations [13].

## 2. Cell-Autonomous Regulatory Mechanisms of MMC Specification

The establishment of MMC fate depends first and foremost on cell-autonomous regulatory networks. These networks center on core transcription factors and cell-cycle regulators, integrating hormonal signals and epigenetic modifications to ensure that a single L2-layer cell correctly initiates the germline developmental program (Figure 2).

*SPOROCYTELESS/NOZZLE* (SPL/NZZ) encodes a nuclear-localized protein and serves as the most critical “master switch” for MMC specification [14,15]. Genetic evidence demonstrates that *SPL/NZZ* acts cell-autonomously in the L2 layer to directly activate the MMC fate determination program. In *spl/nzz* mutants, ovules fail to form an MMC, resulting in complete female sterility, indicating that *SPL/NZZ* is essential for MMC specification.

*WUSCHEL* (*WUS*) is another key transcription factor that is transiently expressed in the MMC precursor cell (the archesporial cell). WUS expression is tightly repressed by *RETINOBLASTOMA-RELATED 1* (*RBR1*); *wus* mutants fail to form an MMC, whereas loss of *RBR1* function leads to ectopic activation of WUS and the formation of multiple MMC-like cells [16]. RBR1 directly binds to the *WUS* promoter and restricts the WUS expression domain through transcriptional repression. Under RBR1-mediated restriction, WUS further indirectly activates expression of its downstream target genes *WINDHOSE1* (*WIH1*) and *WINDHOSE2* (*WIH2*), which encode secreted small peptides that convey regulatory signals to surrounding companion cells to collectively promote megasporogenesis [17]. The precise spatial control of the WUS expression domain is a critical prerequisite for ensuring that only a single L2-layer cell acquires MMC fate.

Notably, SPL/NZZ and WUS engage in complex regulatory interactions. SPL/NZZ forms a complex with MADS-box transcription factors (such as *SEP3* and *STK*) to bind CArG-box motifs in target genes, thereby inducing *WUS* expression in the L2 layer; within the L2 layer itself, SPL/NZZ can directly activate the MMC fate program [8,17]. When mutations in the RNA-directed DNA methylation (RdDM) pathway cause ectopic expression of *SPL/NZZ* to expand into more nucellar cells, multiple MMC-like cells appear, further confirming that precisely restricting the spatial expression of SPL/NZZ is critical for MMC singularity.

Cell-cycle regulators play a key role in restricting MMC number. Cell-cycle progression is tightly coupled to chromatin status; indeed, cell-cycle transition may act as a geometry- and thermodynamics-dependent chromatin-state switch during early ovule primordium development, as supported by three-dimensional in situ cytological observations in ovule tissues [18]. Future application of spatial transcriptomics and in situ proteomics to ovule primordia will be required to identify the specific chromatin remodelers, cyclins, and CDK inhibitors that directly link nuclear geometry to MMC fate commitment. KIP-RELATED PROTEINs *(KRPs*, also known as *ICKs*) are cyclin-dependent kinase (CDK) inhibitors that suppress CYCLIN-DEPENDENT KINASE A;1 (*CDKA;1*) activity, thereby promoting RBR1 accumulation and blocking cell-cycle progression [16,19].

The temporal and hierarchical relationship between the SPL/NZZ-WUS transcriptional axis and the KRP-CDKA;1-RBR1 cell-cycle checkpoint is emerging as a critical question. Current evidence supports a model in which SPL/NZZ acts as the primary fate determinant, initiating the MMC program in the L2 layer, whereas RBR1 functions as a gatekeeper that restricts this fate to a single cell by repressing WUS expression. Loss of RBR1 leads to ectopic WUS activation and supernumerary MMCs even in the presence of SPL/NZZ, indicating that RBR1 acts downstream of or in parallel to SPL/NZZ to limit the number of cells that adopt MMC identity [16]. Temporally, SPL/NZZ expression precedes RBR1-mediated restriction, suggesting an initial “competence” phase followed by a “refinement” phase during which the single MMC is selected from a field of potential precursors. The KRP-CDKA;1 module, by promoting RBR1 accumulation, serves as a rheostat that couples cell-cycle progression to germline fate commitment [20].

Analysis of the *ick/krp* septuple mutant has revealed the redundant function of KRPs in ensuring formation of a single MMC and a single functional megaspore. In the septuple mutant, most ovules form multiple MMCs, and both callose deposition patterns and DMC1 immunostaining confirm that these extra MMC-like cells are capable of entering meiosis and producing multiple functional megaspores [20]. RBR1 is a key negative regulator of cell-cycle progression that prevents formation of extra MMCs by directly repressing *WUS* transcription. Loss of RBR1 function leads to aberrant activation of *WUS* expression, which in turn promotes multiple cells to acquire MMC fate. The KRP–CDKA;1–RBR1 regulatory pathway constitutes an important cell-autonomous mechanism for restricting MMC number, cooperating with hormonal signaling and epigenetic regulatory pathways to ensure establishment of the “one ovule, one megaspore mother cell” principle [16].

Auxin signaling during MMC specification is regulated through a complex dual mechanism. SPL/NZZ shapes the auxin landscape via a dual-mode regulatory module. First, it facilitates *PIN1*-mediated polar auxin transport, which channels auxin from its primary sporophytic sources—the chalaza and funiculus where *TAA1* is expressed—toward the distal nucellus [21]. This enhancement of PIN1-dependent flux occurs indirectly, largely through repressing *ANT* to relieve its inhibitory effect on PIN1 transcription [22]. Second, SPL/NZZ restrains local auxin-biosynthesis capacity within the nucellar apex, possibly by modulating the activity of *YUC-family* biosynthetic genes. This coordinated action suggests that the local auxin maximum at the nucellar apex is generated mainly by directional transport from a spatially defined sporophytic auxin pool rather than by de novo synthesis within the nucellus itself [23]. While additional local biosynthesis via YUC-family enzymes may contribute, the prevailing evidence indicates that the steep gradient required for MMC confinement is established primarily by transport from these defined sporophytic sources. Such a steep gradient is functionally critical for confining MMC specification to a single precursor cell. Nevertheless, the molecular nature of this spatially confined auxin-maximum domain remains incompletely resolved. Auxin accumulation at the nucellar apex could result from multiple non-mutually exclusive inputs: diminished auxin efflux, perturbed auxin canalization flow, or enhanced local auxin biosynthesis mediated by TAA1-TAR-YUC-family enzymes. Collectively, these auxin-related inputs modulate MMC differentiation, a cellular process encompassing chromatin-state reprogramming [22], nuclear morphological remodeling, and lytic-vacuole biogenesis within the archesporial-cell lineage [24].

MicroRNA160 (miR160) and its target gene *AUXIN RESPONSE FACTOR 17* (*ARF17*) constitute a core regulatory circuit for MMC specification. miR160 controls MMC formation by negatively regulating ARF17 expression. In the miR160 loss-of-function mutant *foc* (*floral organs in carpels*), ARF17 protein levels are abnormally elevated, leading to the formation of multiple megaspore mother cell-like (MMC-like) cells [25]. ARF17 protein is predominantly localized in the MMC, and its expression is precisely controlled by miR160 to ensure formation of a single MMC. The miR160–ARF17 module also modulates the expression domain of PIN1, thereby influencing establishment of the auxin maximum at the nucellar apex [1]. ARF17 genetically interacts with SPL/NZZ: overexpression of ARF17 in the *spl* mutant partially rescues MMC formation, indicating that ARF17 promotes MMC specification by modulating SPL/NZZ function. Concurrently, SPL/NZZ also influences PIN1 expression, forming a positive feedback regulatory loop that ensures correct specification of a single MMC [22].

*WIH1* and *WIH2* encode plant-specific small peptides whose functions are associated with RAB GTPASE HOMOLOG A1 (RABA1). WIH peptides may recruit RABA1-modified vesicles to influence polar localization of the auxin transporter PIN1, thereby regulating auxin accumulation in the nucellar epidermis [17]. Genetic evidence indicates that WIH1/WIH2 act synergistically with functionally related proteins such as *TORNADO2* (*TRN2*) to regulate megasporogenesis [26].

Brassinosteroids (BRs) are locally biosynthesized within ovule primordium tissues; BR-hormone distribution shows spatial heterogeneity across nucellar and integument cell layers. Within the MMC lineage itself, BR signaling primarily functions to prime chromatin states that reinforce fate commitment. The BZR1 transcription factor family—including BZR1, BES1, and their homologs BEH1–BEH4—translocates into the nucleus upon dephosphorylation and modulates expression of genes involved in miRNA processing and chromatin remodeling [27,28]. Notably, the SWR1-SDG2 module cooperates with BZR1 to influence H2A.Z deposition at regulatory regions of target genes, thereby contributing to the epigenetic stabilization of MMC fate [29,30]. The non-cell-autonomous function of BR signaling in lateral inhibition via the EPFL–ERECTA pathway is discussed in Section 3 and Section 5.

Gibberellin (GA) and cytokinin (CK) signaling antagonistically regulate MMC specification. Hyperactivation of GA signaling leads to increased numbers and abnormally enlarged MMCs, whereas CK signaling is both required for MMC specification and restricts multiple cells at the distal end of the ovule primordium from acquiring MMC fate. Mechanistically, GA negatively regulates CK action by promoting degradation of the type-B response regulators *ARR1/10/12*, while the vacuolar sorting protein SHBY antagonizes GA signaling by stabilizing ARR1/10/12 proteins [31,32]. Moreover, cytokinin signaling, via its receptors *AHK2*, *AHK3*, and *AHK4/CRE1*, promotes expression of SPL/NZZ and WUS at the nucellar apex, thereby providing permissive signals for correct MMC positioning [26]. Beyond their reciprocal antagonism, GA and CK signaling also crosstalk with auxin-gradient patterning during MMC specification. Cytokinin signaling transcriptionally modulates PIN-family auxin-transporter abundance to sculpt the apical auxin-maximum domain. By contrast, elevated GA signaling may interfere with auxin-biosynthesis or polar-transport machinery, consequently perturbing spatial auxin gradients and altering MMC-cell number and size. Collectively, these multilayered hormonal interactions converge on the auxin biosynthesis module and the transport modules to establish the local auxin gradient that underpins accurate MMC positioning [21].

Deposition of the histone variant H2A.Z plays an important role in MMC fate regulation. The SWR1 complex mediates H2A.Z deposition at promoter regions of target genes, with ARP6 serving as a subunit of this complex. In the *arp6 er-119 sdg2* triple mutant, multiple cells in the nucellar primordium acquire MMC identity and enter meiosis [31]. SDG2-mediated H3K4me3 histone modification may participate in regulating this process. The BZR1 transcription factor family directly activates *HYL1*, *DCL1*, and *SE* gene expression, a process regulated by the SWR1–SDG2–ER signaling module. In the *arp6 er-119 sdg2* mutant and the BZR1 loss-of-function mutant *sex-1*, H2A.Z deposition at nucleosome regions near the transcription start sites (TSS) of *HYL1*, *DCL1*, and *SE* is significantly reduced, indicating that the SWR1–SDG2–ER module cooperates with BZR1 to promote H2A.Z deposition and activate expression of these genes, thereby ensuring the singularity of MMC formation and its subsequent entry into meiosis [31,32].

The RNA helicase *MNEME* (*MEM*) is another epigenetic regulator important for MMC fate maintenance. MEM belongs to the DEAD-box family of ATP-dependent RNA helicases and is specifically enriched in the MMC [3]. In *mem* mutants, multiple MMC-like cells are formed, and higher-order chromatin structure and distribution of LIKE HETEROCHROMATIN PROTEIN 1 (LHP1) in female gametophyte nuclei are affected, indicating that loss of MEM function leads to epigenetic perturbations. The phenotype of *mem* mutants resembles that of mutants in small RNA and DNA methylation pathways, suggesting that MEM may restrict germline fate by cooperating with these epigenetic pathways. Importantly, *mem* mutants exhibit features of apospory, where the female gametophyte initiates development from two non-sister cells, indicating that multiple cells have acquired the ability to enter meiosis and produce functional megaspores [3].

DNA methylation homeostasis is a core mechanism for maintaining a single MMC. Systematic analyses have revealed that either a decrease or an increase in any of the three types of DNA methylation (mCG, mCHG, and mCHH) leads to an abnormal increase in MMC-like cells, and restoring methylation balance rescues the mutant phenotype. Through single-cell quantitative DNA methylation analysis, the study further revealed that in the MMC precursor cell, local mCHH levels decline prior to expression of the MMC marker gene KNUCKLES (KNU). Disrupting DNA methylation regulation causes mCHH levels in the MMC and its neighboring cells to converge, even though these neighboring cells are normally destined to differentiate into somatic cells. Temporally, the level of the de novo DNA methyltransferase DRM2 decreases before KNU expression, whereas the level of the DNA glycosylase DEMETER (DME) declines only after KNU expression [33]. Together, these results indicate that the key to maintaining a single MMC lies in precise spatiotemporal regulation of DNA methylation within the MMC lineage, rather than a simple increase or decrease in overall methylation levels [34].

DEMETER (DME) is a DNA glycosylase that plays a crucial demethylation role in plant reproductive development. DME is specifically expressed in the central cell and regulates endosperm development by mediating DNA demethylation to activate the imprinted gene *MEDEA* (*MEA*) [35,36]. Recent studies have further revealed that DME also functions in MMC specification, and precise regulation of its expression level is essential for maintaining appropriate DNA methylation balance in the MMC region [34].

The RNA-directed DNA methylation (RdDM) pathway plays a key role in restricting MMC number through cell-autonomous epigenetic stabilization. ARGONAUTE 9 (AGO9) is a core component of the RdDM machinery and, by binding 24-nucleotide (nt) small interfering RNAs (siRNAs), silences transposable elements (TEs) in the nucellus, thereby controlling germline cell specification [37]. Approximately 50% of ago9 mutant ovules exhibit supernumerary MMCs, indicating that loss of AGO9 function leads to aberrant acquisition of MMC fate by neighboring cells [5,38]. The mobile nature of AGO9-associated siRNAs and their non-cell-autonomous silencing of germline genes in adjacent somatic cells are discussed in Section 3 and Section 5.

RNA-dependent RNA polymerase 6 (*RDR6*) and *SUPPRESSOR OF GENE SILENCING 3 (SGS3*) participate in trans-acting siRNA (tasiRNA) biogenesis, and mutations in these genes result in the formation of multiple MMCs. The tasiRNA pathway acts in parallel with the AGO9-mediated RdDM pathway to jointly restrict MMC number [37,39]. *TAS3*-derived tasiRNAs suppress companion cell acquisition of MMC fate by restricting *ARF3* expression in L2-layer cells [40].

The MADS-box transcription factor *SEEDSTICK* (*STK*) restricts MMC number by regulating expression of RdDM components. STK directly binds the regulatory regions of *AGO9* and *RDR6* and promotes their expression in cells of the lower L1 layer of the nucellar epidermis, thereby epigenetically repressing ectopic expression of *SPL/NZZ* in nucellar cells through the RdDM pathway [8]. In *stk* mutants, *SPL/NZZ* expression is upregulated while *RDR6* and *AGO9* expression is reduced, leading to the formation of multiple MMC-like cells. This regulatory loop reveals a molecular mechanism by which transcription factors and small RNA pathways coordinately regulate MMC fate.

In *Arabidopsis*, CLASSY (*CLSY*) family proteins (*CLSY1–4*) achieve locus-specific RdDM regulation independently of histone modification (H3K9me2) by interacting with different subunits of RNA polymerase IV (Pol IV) [41]. This finding uncovers a new layer of RdDM pathway regulation and provides important insights into mechanisms of DNA sequence-specific recognition during plant development.

KNUCKLES (KNU) encodes a C2H2-type zinc finger protein. Early studies revealed its role in development of the basal structure of the gynoecium and floral meristem termination [42]. Subsequently, KNU was detected to be specifically expressed in the MMC and is now widely used as a molecular marker for MMC identity [42]. However, the biological function of KNU during megaspore mother cell specification remains unclear and awaits further investigation.

The core transcriptional module dominated by SPL/NZZ and WUS serves as the central regulatory hub for female germline specification and is tightly modulated by multiple hormonal signaling pathways. Auxin signaling contributes positively to MMC differentiation through ARF17-mediated regulation of SPL/NZZ activity, while miR160 maintains proper ARF17 dosage to ensure single-MMC singularity [25]. Cytokinin acts as a core permissive signal that directly promotes the expression of SPL/NZZ and WUS at the nucellar apex via AHK2/3/4 receptors, establishing the spatial competence zone for germline initiation [26]. In contrast, GA functions antagonistically to CK by facilitating the degradation of type-B ARR transcription factors, thereby dampening CK-dependent germline-promoting signaling [29]. Distinctly, BR signaling within the MMC lineage primarily operates to reinforce chromatin states through BZR1-mediated transcriptional modulation and H2A.Z deposition [31,32]. Collectively, these hormone pathways converge on the SPL/NZZ–WUS regulatory axis through distinct modes—direct transcriptional activation (CK), indirect genetic modulation (auxin), signal antagonism (GA), and chromatin-level reinforcement (BR)—thereby translating multilayered hormonal spatial gradients into a robust binary cell-fate decision that guarantees accurate MMC specification.

## 3. Non-Cell-Autonomous Signals from Somatic Cells to the MMC

MMC specification depends not only on cell-autonomous programs but also on precise regulation by surrounding somatic cells. Signals originating from the L1 epidermis, the chalazal end, and the integuments provide positional information and impose constraints on a single L2-layer cell to ensure it acquires MMC fate at the correct location [43] (Figure 2).

Although SPL/NZZ functions cell-autonomously in the L2 layer (as described in Section 2), its expression at the nucellar apex is predominantly localized to L1-layer cells, and it can regulate MMC differentiation in the underlying L2 layer in a non-cell-autonomous manner [8]. SPL/NZZ in the L1 layer promotes MMC formation by inducing *WUS* expression in the L2 layer. This cross-layer signal transmission ensures that MMC specification initiates at the correct position—the distal L2 layer. Consistent with the cell-autonomous requirement for *SPL/NZZ* restriction (Section 2), RdDM pathway mutations cause ectopic *SPL/NZZ* expansion and supernumerary MMC-like cells, underscoring that spatial confinement of SPL/NZZ is essential for singularity.

*ARGONAUTE 9* (*AGO9*) is predominantly localized in the cytoplasm of somatic companion cells in the L1 layer of the nucellar epidermis [37]. Notably, the transcriptional regulation and localization pattern of AGO9 vary among different *Arabidopsis* ecotypes, and transient expression can be detected in the MMC in certain ecotypes [44]. AGO9 preferentially binds 24-nt siRNAs, which can move from the L1 layer into the adjacent L2 layer and establish DNA methylation in companion cells via the RdDM pathway, thereby silencing expression of germline program genes such as *SPL/NZZ* [37]. This non-cell-autonomous mobile signal serves as a key barrier to maintaining MMC singularity; approximately 50% of ovules in *ago9* mutants contain multiple MMCs [5].

As discussed in Section 2, CK signaling from sporophytic tissues generates a permissive gradient that upregulates *SPL/NZZ* and *WUS* in the distal L2 layer, thereby defining the competence zone for MMC specification. [26]. CK signaling antagonizes GA signaling: CK is both required for MMC specification and restricts multiple cells at the distal end of the ovule primordium from acquiring MMC fate. This hormonal antagonism provides concentration-dependent positional information for MMC specification [45,46].

The signaling module composed of EPIDERMAL PATTERNING FACTOR-LIKE (*EPFL*) family small peptides and ERECTA family (*ERf*) receptor kinases represents one of the core pathways regulating MMC specification and integument development. The ERECTA family receptor kinases include *ERECTA* (*ER*), *ERECTA-LIKE 1* (*ERL1*), and *ERECTA-LIKE 2* (*ERL2*), which are expressed in L1-layer cells of the nucellar epidermis, the inner integument, the chalazal region, and the funiculus [31].

The *Arabidopsis* EPFL family contains 11 members, among which *EPFL1*, *EPFL2*, *EPFL4*, and *EPFL6* play redundant regulatory roles in ovule development. Single and double mutants exhibit normal fertility, whereas triple mutants show reduced fertility, and the *epfl1/2/4/6* quadruple mutant is completely sterile [28]. EPFL1, EPFL4, and EPFL6 are mainly expressed in nucellar epidermal cells of the ovule primordium, an expression pattern similar to that of the ERf receptors [28].

Two distinct phenotypes are observed in the *epfl1/2/4/6* quadruple mutant: first, in some ovules, cells become excessively enlarged and acquire MMC identity, but these cells fail to develop into functional MMCs capable of completing meiosis; second, some ovules fail to form any MMC at all [28]. These results indicate that EPFL family small peptides are the key ligands of the ERf receptor-mediated signal that restricts MMC specification.

Upon receiving EPFL ligand signals, ERf receptor kinases activate multiple downstream signaling pathways. First, EPFL–ERf signaling activates the BZR1 transcription factor family via the BR signal transduction pathway, which in turn transiently activates expression of WRKY23 [27] and NSN1 [28], thereby inhibiting surrounding somatic cells from acquiring MMC fate; however, this process does not directly regulate entry into meiosis [47].

Second, the SWR1–SDG2–ER module promotes accumulation of BZR1 protein at promoter regions of target genes, upregulating expression of key components of the miRNA processing pathway, such as *SE*, *HYL1*, and *DCL1*. Activation of these genes ensures that only a single cell in the ovule primordium acquires MMC fate and enters meiosis. Genetic analysis shows that *er-119 hyl1* double mutant ovules exhibit multiple MMCs capable of undergoing meiosis, and these ovules contain two additional populations of functional megaspores, further validating the functional importance of this regulatory pathway [31].

In parallel, SOMATIC EMBRYOGENESIS RECEPTOR-LIKE KINASES (SERKs) act as co-receptors in ERf-mediated signal transduction. *SERK1*, *SERK2*, and *SERK3* interact with ERf, and this interaction depends on EPFL family small peptides [48]. The *serk1/2/3* triple mutant exhibits integument development arrest and abnormal embryo sac phenotypes similar to those of *er erl1/2*, indicating that SERKs are key components of the ERf–EPFL signaling pathway [31].

*PIN-FORMED 1* (*PIN1*) is a key carrier protein regulating polar auxin transport during MMC specification. PIN1 exhibits basal–apical polar localization in L1-layer cells of the nucellar epidermis, transporting auxin from the chalazal region and the funiculus toward the distal nucellus and establishing a local auxin maximum at the nucellar apex. This process is precisely controlled by the miR160–ARF17 signaling pathway [1,22].

In *spl/nzz* mutants, PIN1 expression in the nucellar epidermis and the chalaza nearly disappears, resulting in failure to establish the auxin maximum at the nucellar apex [22]. When *PIN1* expression is driven by the *SPL/NZZ* promoter in the *spl/nzz* mutant background, normal auxin maximum and MMC differentiation are restored, demonstrating that SPL/NZZ determines MMC fate by directly activating *PIN1* expression [22].

Treatment of wild-type inflorescences with the auxin transport inhibitor N-1-naphthylphthalamic acid (NPA) leads to production of MMC-like cells in approximately 62.6% of ovules, further confirming the importance of PIN1-mediated polar auxin transport in restricting MMC number [1].

The AUXIN RESPONSE FACTOR (ARF) transcription factor family translates auxin signals into changes in gene expression. ARF17 is predominantly expressed in the MMC, the chalazal region, and the funiculus, and its protein level is precisely controlled by miR160. ARF17 protein is detected exclusively in the MMC, while only faint fluorescent signals are observed in the chalazal region and the funiculus [1].

A low level of ARF17 protein is essential for MMC formation. In the *foc* (miR160 loss-of-function) mutant, ARF17 protein levels are abnormally elevated; likewise, MMC-like cells are observed in transgenic plants expressing miR160-resistant ARF17 (*pARF17::mARF17*). Conversely, MMC formation is impaired in the *arf17* mutant, and approximately 74% of ovules in the *arf17 spl-3* double mutant fail to form an MMC [1].

*ARF10* is another ARF transcription factor that plays a critical role in cells surrounding the MMC. In wild-type plants, ARF10-GFP expression is restricted to cells surrounding the MMC. However, in miR160-insensitive transgenic lines, ARF10 becomes distributed throughout the ovule, leading to formation of multiple functional megaspores. This indicates that ARF10 maintains a low-level signal in cells surrounding the MMC, thereby preventing these cells from differentiating into MMC-like cells [49].

SPL/NZZ, ARF17, and PIN1 constitute a precise regulatory network that ensures correct specification of a single MMC. Within this network: (1) SPL/NZZ promotes *PIN1* expression by repressing expression of chalazal genes such as *ANT* [22]; (2) PIN1-mediated auxin transport establishes an auxin maximum at the nucellar apex [1]; (3) auxin induces *ARF17* expression, while miR160 precisely restricts ARF17 protein levels [1]; (4) ARF17 genetically interacts with SPL/NZZ to promote MMC differentiation [1]; (5) SPL/NZZ further influences auxin distribution by modulating the expression domain of *PIN1* [1,22]. This regulatory network integrates multiple layers of mechanisms—including transcription factor regulation, miRNA-mediated translational repression, hormone signal transduction, and polar transport—forming a complex regulatory system that ensures the principle of “one ovule, one megaspore mother cell.”

*TPD1/MAC1* (TAPETUM DETERMINANT 1/MULTIPLE ARCHESPORIAL CELLS 1) is a cysteine-rich secreted protein that functions as a ligand for the *EXS1/EMS1* receptor. TPD1/MAC1 is secreted from germ cells (including the MMC and its precursor cells) and acts on surrounding somatic cells, conveying a non-cell-autonomous inhibitory signal to restrict these neighboring cells from acquiring germline fate [12,13,50]. In maize, loss of MAC1 function leads to formation of multiple germ cells in both anthers and ovules, indicating that this signaling module plays a conserved restrictive role in both male and female germline development [13].

Transmission of the TPD1/MAC1 signal depends on direct cell-to-cell contact or short-range diffusion. Upon ligand-receptor binding, downstream signal transduction pathways are likely activated, thereby regulating expression of target genes and influencing the developmental fate of surrounding cells. RNA-seq analysis has shown that overexpression of TPD1 alters expression of auxin signaling pathway genes and core cell-cycle genes in the ovule [51]. However, the specific target genes and downstream effectors of TPD1/MAC1 in ovule development remain to be further investigated.

In addition to the EPFL family and TPD1/MAC1, other cysteine-rich small peptides may also participate in MMC-related intercellular communication. RAPID ALKALINIZATION FACTOR (RALF) family peptides and LURE-type peptides play diverse roles in plant reproductive development. RALF peptides regulate cell elongation and defense responses via the FERONIA (*FER*) receptor kinase [52], while LURE-type peptides act as pollen tube attractants to mediate fertilization [53] Although the direct functions of RALF and LURE-type peptides in MMC specification have not yet been demonstrated, their receptor FERONIA is known to be expressed in the integuments and the filiform apparatus of the ovule [54], suggesting that these small peptides may participate, in a redundant or subtle manner, in communication between the MMC and surrounding cells through as-yet-unidentified mechanisms.

In wild-type ovules, the expression domains of key regulatory factors are precisely spatially separated: SPL/NZZ is mainly expressed in apical cells of the L1 layer and non-cell-autonomously regulates MMC differentiation in the underlying L2 layer; WUS expression is restricted to the MMC precursor cell through RBR1-mediated repression; and the inhibitory factors WRKY23 (and WRKY28) are expressed in companion cells (L2- layer) rather than in the nucellar center. This precise spatial separation of key regulatory factors ensures that only a single L2 cell positioned at the “right place” can receive sufficient permissive signals and initiate the MMC program. An increase in MMC number is observed when ectopic expression of SPL/NZZ expands into more nucellar cells due to mutations in the RdDM pathway [8], or when multiple MMC-like cells form in the *rbr1* mutant, confirming the importance of this spatial separation mechanism.

How are these diverse non-cell-autonomous signals, which originate from the L1 epidermis, the chalazal end, and the integuments, integrated to ensure that only a single L2 cell acquires MMC fate? Current evidence suggests a model in which permissive and restrictive signals are spatially separated and temporally coordinated. Permissive signals (auxin from the chalaza via PIN1 transport [22]; cytokinin from sporophytic tissues [26]) converge at the nucellar apex to define a “competence zone,” whereas restrictive signals (AGO9-RdDM derived from the L1 epidermal layer [37]; EPFL-ERECTA-BZR1 cascades in surrounding epidermal cells [28]; gibberellin originating from sporophytic tissues [29]) act as lateral inhibitory cues to exclude adjacent subepidermal cells from this germline-competent domain. The primary integration hub of these divergent signals is the *SPL/NZZ-WUS* transcriptional regulatory node, which receives stimulatory inputs from auxin (mediated by ARF17) and cytokinin (mediated by AHK histidine kinase receptors), while its expression is epigenetically repressed by RdDM silencing in neighboring somatic cells [22]. This “spatial coincidence” working model posits that only a single cell positioned at the overlapping intersection of multiple permissive hormonal gradients and outside the effective range of all restrictive inhibitory signals can fully initiate the MMC developmental program. Nevertheless, the precise molecular details describing how these distinct signaling cascades converge onto shared chromatin targets to shape cell-fate decisions remain incompletely elucidated.

## 4. Mechanisms by Which Neighboring Somatic Cells Maintain Their Fate and Restrict MMC Number

While the MMC initiates its own fate program, surrounding somatic cells must establish stable molecular barriers to prevent themselves from aberrantly acquiring germline characteristics. This process involves multi-layered events including epigenetic remodeling, transcription factor network reprogramming, and cell wall modification (Figure 2).

One of the earliest changes that occur in neighboring cells upon receiving inhibitory signals is dynamic adjustment of DNA methylation levels. In wild-type ovules, mCHH levels are lower in the central MMC region than in surrounding cells, and this difference is essential for maintaining distinct fates of the MMC and its neighbors. When DRM2 (de novo DNA methyltransferase) function is lost, mCHH levels in the MMC and its neighboring cells converge, causing neighboring cells to fail to maintain their normal somatic fate and instead aberrantly acquire MMC-like characteristics [34]. Reintroducing DRM2 or knocking down DNA demethylase (DME) in neighboring cells both rescues the supernumerary MMC phenotype of the drm1 drm2 double mutant, confirming that DNA methylation homeostasis in surrounding cells is the basis for their correct developmental response [34]. Notably, DME also functions in MMC specification, and precise regulation of its expression level is essential for maintaining appropriate DNA methylation balance in the MMC region [34].

The RdDM pathway plays a central role in maintaining DNA methylation patterns in neighboring cells. AGO9 is specifically expressed in L1 epidermal companion cells, and its protein is not localized in the MMC. AGO9 binds 24-nt siRNAs and directs DRM2 to establish and maintain DNA methylation in companion cells, thereby silencing genes associated with germline fate (such as SPL/NZZ) in somatic cells [8,37]. In ago9 or rdr6 mutants, SPL/NZZ is ectopically expressed in neighboring cells, causing them to aberrantly initiate the germline developmental program [8]. Thus, the RdDM pathway serves as a mobile epigenetic barrier that reinforces somatic identity in cells adjacent to the MMC.

A fundamental unresolved conceptual question is whether the three major classes of epigenetic reprogramming during ovule development—dynamic DNA methylation remodeling, H2A.Z histone variant deposition, and mobile small RNA pathway activation—act as upstream instructive drivers of MMC specification or merely downstream secondary consequences of cell-fate commitment. Current evidence supports a nuanced dual-function model: a subset of epigenetic alterations function as causal fate-specifying cues, while others serve only to stabilize already established germline identity. The locus-specific decline in mCHH methylation within the MMC precursor occurs prior to detectable KNU transcriptional activation, representing an early permissive epigenetic shift that licenses germline gene expression; genetic disruption of this methylation–demethylation balance in drm1 drm2 double mutants readily triggers supernumerary MMC formation [34]. In parallel, AGO9-dependent RdDM signaling originating from L1 epidermal cells exerts a causal inhibitory function by epigenetically silencing SPL/NZZ in subepidermal somatic neighbors, strictly restricting germline competence to a single cell per ovule [8]. In contrast, H2A.Z chromatin deposition and permissive histone marks such as H3K4me3 predominantly accumulate after MMC fate is initiated, functioning as reinforcing epigenetic locks that stabilize germline transcriptional programs and prevent fate reversion [31]. Definitive discrimination between causal instructive epigenetic signals and passive fate-correlated chromatin remodeling will require temporally controlled, cell-type-specific epigenetic perturbation systems that modify individual marks within narrow developmental windows.

Upon receiving signals, neighboring cells undergo changes in histone modifications, which are important hallmarks of chromatin remodeling and gene expression reprogramming. Deposition of the histone variant H2A.Z in neighboring cells is regulated by the SWR1 complex. Loss of function of ARP6, a subunit of the SWR1 complex, leads to reduced H2A.Z deposition at promoter regions of target genes, thereby affecting normal gene expression [31,55]. SDG2-mediated H3K4me3 modification cooperates with the SWR1 complex to activate expression of BZR1 transcription factors and their downstream target genes in neighboring somatic cells. BZR1 directly activates expression of miRNA processing factor genes such as SE, HYL1, and DCL1, and activation of these genes in neighboring cells is essential for restricting MMC number. In addition, BZR1 directly activates expression of WRKY23 and NSN1 [27,28], and these transcription factors and signaling proteins are specifically expressed in neighboring cells to suppress their acquisition of MMC fate. In the arp6 er-119 sdg2 triple mutant, H2A.Z deposition at promoter regions of genes such as SE, HYL1, and DCL1 is reduced, leading to downregulation of their expression and consequently formation of multiple MMCs [31].

In cell-cycle regulation, RBR1 forms a complex with histone deacetylases (HDACs) and participates in transcriptional repression of cell-cycle-related genes [16]. However, whether RBR1 directly represses transcriptional activity of the *WUS* promoter by recruiting HDACs, and whether phosphorylation and acetylation states of histone H1 undergo dynamic changes during MMC specification, still lack direct experimental evidence and await further investigation.

Upon receiving MMC-derived signals, small RNA-mediated gene silencing pathways are activated in neighboring cells, serving as a crucial mechanism for maintaining somatic cell fate. RDR6 and SGS3 participate in tasiRNA biogenesis, and mutations in these genes lead to formation of multiple MMCs [39]. The tasiRNA pathway acts in parallel with the AGO9-mediated RdDM pathway to jointly restrict MMC number [37,39]. TAS3-derived tasiRNAs suppress acquisition of MMC fate by companion cells by restricting ARF3 expression in L2-layer cells [39,40].

STK promotes expression of *AGO9* and *RDR6* in lower L1-layer cells by directly binding to their regulatory regions. AGO9 preferentially binds 24-nt siRNAs and represses expression of germline genes such as *SPL/NZZ* in somatic cells at the transcriptional level through the RdDM pathway [8]. This small RNA-mediated gene silencing mechanism constitutes a molecular barrier that allows somatic cells to maintain their own fate and prevents aberrant acquisition of germline characteristics.

The tasiRNA pathway restricts expression of ARF3 (AUXIN RESPONSE FACTOR 3) in the lower nucellus through tasiRNAs produced from the *TAS3* locus. ARF3 expression in cells surrounding the MMC is tightly restricted, preventing these cells from acquiring MMC fate. When the tasiRNA pathway is compromised, ARF3 is ectopically expressed in neighboring cells, leading to MMC-like cells [39,40].

Upon receiving signals, WRKY transcription factor family members are activated in neighboring cells, where they function to repress germline fate. WRKY23 is a direct target gene of the BZR1 transcription factor family and is specifically expressed in subepidermal somatic cells (L2 somatic cells) surrounding the MMC [27]. Upon EPFL–ERECTA-mediated signal perception in the L1 epidermis (see Section 3), BR signaling activates BZR1 via the BRI1 receptor; BZR1 then enters the nucleus and binds to the WRKY23 promoter to activate its transcription. Expression of WRKY23 ensures that neighboring cells maintain somatic fate and do not acquire MMC identity.

WRKY28 is another WRKY transcription factor that exerts an inhibitory function in adjacent subepidermal cells. Expression of WRKY28 is activated by KLUH/CYP78A5 (KLU) [47], a member of the cytochrome P450 family expressed in the inner integument primordium, which non-cell-autonomously activates *WRKY28* expression in neighboring cells through SWR1 complex-mediated chromatin remodeling [56]. The expression pattern of WRKY28 is complementary to that of WRKY23, and together they constitute a dual safeguard mechanism that inhibits neighboring cells from acquiring MMC fate.

The *wrky28* mutant exhibits a phenotype of multiple MMC-like cells, similar to that of the *wrky23* mutant, indicating that these two transcription factors are functionally redundant. Notably, the multiple MMC-like cell phenotype in the *qui-1* mutant is more severe than that in the *wrky23* and *nsn1* single mutants, and some of the extra MMCs can enter meiosis, a phenomenon not observed in *wrky23* and *nsn1* mutants, suggesting that BZR1 may regulate other unidentified target genes to further restrict MMC entry into meiosis [31].

NUCLEOSTEMIN-LIKE 1 (NSN1) is another important target gene of the BZR1 transcription factor family and encodes a nucleolar GTP-binding protein [31]. Expression of NSN1 in neighboring cells is regulated by the BR–BZR1 signaling pathway, and its function is to restrict these cells from acquiring MMC fate [27].

In animals, NSN1 homologs are known to participate in regulating stem cell proliferation and ribosome biogenesis. In *Arabidopsis*, NSN1 acts synergistically with WRKY23 to suppress acquisition of MMC fate in neighboring cells, as the *nsn1 wrky23* double mutant exhibits a more severe multiple-MMC phenotype than either single mutant. Although the *nsn1* single mutant also displays a multiple-MMC phenotype, its effect is relatively mild, suggesting the possible existence of other functionally redundant genes or compensatory mechanisms [31].

Neighboring somatic cells sense and respond to multiple classes of intercellular signals originating from the L1 epidermal layer and the central MMC precursor via multiple ligand-receptor and epigenetic mobile RNA systems. The ERECTA family receptor kinases (ER, ERL1, ERL2) expressed in L1 epidermal cells recognize secreted EPFL-family small peptides, triggering a downstream signaling cascade that culminates in BZR1-dependent transcriptional activation of WRKY23 and NSN1 in underlying L2 somatic cells to block ectopic germline acquisition [28,31]. In grass crops such as rice, the MMC precursor secretes the TPD1-homologous peptide OsTDL1A, which is perceived by the LRR receptor kinase MSP1 expressed in surrounding somatic companion cells; this ligand-receptor module transmits a conserved non-cell-autonomous inhibitory signal that limits somatic cells from adopting germline fate, and loss-of-function mutants produce supernumerary ovular MMC-like cells [49,57]. In parallel, mobile 24-nt siRNAs loaded into AGO9 protein complexes move from L1 companion epidermal cells inward into the subepidermal L2 layer, establishing repressive DNA methylation at loci including SPL/NZZ through the canonical RdDM pathway [37]. The integration of all three signaling inputs ultimately converges at the chromatin level: repressive epigenetic marks (DNA hypermethylation, H2A.Z histone variant deposition) and permissive somatic histone modifications (such as H3K4me3) cooperatively establish a stable somatic cell transcriptional identity that is refractory to switching toward germline fate.

Callose (β-1,3-glucan) deposition is an important cytological feature of the MMC and meiotically dividing cells. In wild-type ovules, faint and punctate callose deposition can be observed in the cell wall surrounding the MMC before meiosis. After the first meiotic division, strong callose discs or bands appear at the newly formed cell plate, and intense callose deposition is also visible at the micropylar end of the nucellus [20,58].

After the second meiotic division, a second callose band appears at the new cell plate formed by division of the chalazal nucleus, whereas almost no callose is observed at the expected cell plate formed by the micropylar nucleus. This characteristic callose distribution pattern (two major callose bands plus micropylar-directed callose deposition) is a hallmark of completion of meiosis by a single MMC [20].

In mutants that form multiple MMCs, each MMC produces its own characteristic callose deposition pattern. For example, in the *ick/krp* septuple mutant, approximately 17% of ovules contain a single typical callose deposition pattern (originating from a single MMC), about 61% contain two such patterns, and 22% contain three or more. These data indicate that the multiple MMCs in the septuple mutant are capable of completing meiosis [20]. Analysis of the *tramgap* mutant further confirms the reliability of callose deposition as a marker of MMC identity: in *tramgap* mutant ovules containing multiple enlarged cells, multiple cells exhibit callose deposition, indicating that these cells have all acquired MMC identity [59].

Callose deposition is not only a morphological hallmark of the MMC and meiotically dividing cells, but may also serve important physiological functions. As a cell wall polysaccharide, callose may act as a temporary barrier during meiosis, isolating the meiotic cell from the surrounding somatic environment and preventing external signals from interfering with the meiotic process [60]. At the same time, deposition and degradation of callose may regulate intercellular material transport and signal communication. After completion of meiosis, degradation of callose surrounding the functional megaspore may create the necessary cell wall conditions for its subsequent development into the female gametophyte. However, the specific molecular functions of callose deposition in MMC specification and meiosis still require further investigation.

Neighboring cells maintain cytoplasmic continuity through plasmodesmata, which allow free diffusion of small molecules and signaling substances between cells. However, as development progresses, plasmodesmata between the MMC and surrounding cells are gradually modified, leading to reduced permeability [61]. Callose deposition at plasmodesmata may be one of the causes of this reduced permeability. Such modification of plasmodesmata may help partially isolate the MMC from the surrounding somatic cell environment, creating a relatively independent microenvironment for the MMC to enter meiosis. At the same time, macromolecules such as small RNAs can still be selectively transported through specialized plasmodesmal channels, enabling directional intercellular communication [62].

Upon receiving signals, neighboring cells maintain relatively stable somatic characteristics in terms of cell volume and nuclear morphology, in striking contrast to the enlarged MMC. In wild-type ovules, the MMC exhibits an enlarged cell volume and a prominent nucleus and nucleolus, whereas neighboring cells retain a smaller cell volume and a relatively smaller nucleus [63].

In terms of organelle distribution, nucellar epidermal cells contain abundant chloroplasts, and secretory pathway organelles such as the endoplasmic reticulum and Golgi apparatus also retain their normal distribution and structure. Compared with the MMC, neighboring cells exhibit a lower degree of vacuolization and have denser cytoplasm. The MMC, in contrast, displays formation of a large central vacuole, an important cytological preparation before entering meiosis. The plastids in the MMC remain in an undifferentiated proplastid state, whereas plastids in neighboring somatic cells differentiate into amyloplasts or chloroplasts [64,65].

## 5. Conclusions

Specification of the megaspore mother cell (MMC) in *Arabidopsis* is a key event in female germline development, and its core lies in the establishment and maintenance of the fundamental “one ovule, one megaspore mother cell” principle. In recent years, with the aid of single-cell transcriptomics, high-resolution three-dimensional imaging, gene editing, and epigenomic analyses, the field has achieved systematic breakthroughs in understanding mechanisms of MMC fate determination. This review systematically summarizes research progress in the following three integrated levels.

Cell-autonomous regulatory mechanisms: Studies have revealed the critical roles of the SPL/NZZ–WUS core transcription factor network, the KRP–CDKA;1–RBR1 cell-cycle regulatory pathway, and epigenetic factors such as histone variant H2A.Z deposition and the RNA helicase MEM in restricting MMC number. Additionally, hormonal signals including auxin, brassinosteroids, gibberellins, and cytokinins precisely regulate MMC differentiation and maintenance through elaborate spatiotemporal integration. Non-cell-autonomous signals from somatic cells: Studies have established that diverse signaling molecules originating from the L1 layer and the chalazal end (e.g., SPL/NZZ, AGO9, EPFL family small peptides) act through receptor kinases (e.g., ERf, SERK) and downstream transcription factor networks (e.g., BZR1) to non-cell-autonomously regulate MMC specification in the underlying L2 layer. Among these, the EPFL–ERECTA signaling module and the direct regulation of the auxin network by SPL/NZZ are current research hotspots. Mechanisms by which neighboring cells maintain their fate: Upon receiving signals, neighboring somatic cells undergo dynamic remodeling of DNA methylation levels (especially mCHH), H2A.Z-mediated chromatin state changes, activation of small RNA pathways (RdDM, tasiRNA), and reprogramming of transcription factors such as WRKY23/28 and NSN1, thereby establishing a stable somatic identity barrier. In addition, specific callose deposition and plasmodesmatal modification provide structural isolation for MMC meiosis. In summary, MMC specification is a multi-level, high-precision developmental process coordinately regulated by cell-autonomous programs, non-cell-autonomous positional signals, and lateral inhibition mechanisms.

Despite substantial progress, several key questions persist. The identity of the initial trigger that directs a single L2 cell to adopt MMC fate remains elusive—whether it arises from geometric constraints, mechanical forces (as mentioned in the Introduction regarding MMC expansion-induced tension), or local metabolic states is unknown. How multiple signaling pathways (SPL/NZZ-WUS, ERf-BZR1, auxin, CK, GA, BR) are spatiotemporally integrated within the same cell, and whether a core “decision module” exists, awaits quantitative systems-level modeling. Furthermore, neighboring somatic cells must decode and differentially respond to distinct signals from the L1 epidermis and the MMC, yet the molecular nature of MMC-derived mobile signals remains poorly characterized. Finally, systematic comparative studies in crops (maize, rice, tomato) are needed to determine the conservation versus divergence of regulatory pathways established in *Arabidopsis*.

One of the most pressing unanswered questions is the identity of the earliest molecular event that distinguishes the MMC precursor from its neighboring somatic cells. While several markers—including ovular KNU expression, callose deposition, and local DNA hypomethylation—are associated with the MMC fate, most currently available evidence remains correlative rather than causal. The earliest detectable molecular shift so far reported involves localized chromatin remodeling and transient mCHH hypomethylation at specific genomic loci, as uncovered by recent single-cell ovule methylome profiling. However, distinguishing causal instructive epigenetic signals from passive fate-associated epigenetic remodeling requires temporally resolved, cell-type-specific genetic perturbation tools. The development of inducible CRISPR/dCas9 epigenome-editing systems, which can target regulatory regions such as the SPL/NZZ promoter within narrow developmental windows, provides a promising experimental strategy to dissect whether these epigenetic alterations drive MMC specification or merely arise as downstream consequences of cell-fate commitment.

Single-cell transcriptomics has revolutionized the study of MMC specification by providing unbiased molecular portraits of germline and somatic ovule cell populations. However, several plant-specific and analytical technical limitations must be acknowledged when interpreting these datasets. First, MMCs are extremely rare within ovule tissue—only one precursor cell forms per ovule—and protoplast isolation for single-cell capture introduces stress-induced transcriptional artifacts and biases recovery toward cell populations with more digestible cell walls. Second, the limited total number of captured MMC transcriptomes across biological replicates weakens statistical power, hindering reliable discrimination between biologically intrinsic cell-fate heterogeneity and stochastic transcriptional noise. Third, and most critically, standard scRNA-seq captures only a static transcriptional snapshot at the moment of tissue dissociation, creating an inherent analytical ambiguity: intermediate transcript profiles cannot be definitively assigned either to transitional developmental states along a single differentiation trajectory or to distinct stable somatic subpopulations with unique intrinsic cell fates. Computational temporal inference tools including pseudotime ordering and RNA velocity can partially resolve this ambiguity but remain correlative rather than causal readouts of real developmental progression. Integrative experimental pipelines combining scRNA-seq with genetic lineage tracing and temporally resolved live ovule imaging will be indispensable to disentangle stage-dependent transcriptional shifts from fate-determining gene programs, and to formally distinguish transcriptional changes that actively drive MMC specification from those that arise as secondary downstream consequences of cell-fate commitment.

Direct, time-resolved visualization of MMC specification remains one of the most critical yet technically challenging bottlenecks in female germline research. Unlike accessible shoot and root meristems, ovule primordia are deeply embedded within pistil tissues, making long-term, non-invasive live-cell tracking extremely difficult. Current knowledge of MMC developmental timing largely relies on staged fixed-tissue imaging and 3D morphometric reconstruction. High-resolution geometric analyses in both Arabidopsis and maize have demonstrated that nucellar tissue growth patterns and mechanical geometry spatially prepattern the single MMC positioning prior to its morphological enlargement. These landmark studies successfully resolved tissue-level morphological dynamics but cannot capture continuous single-cell behavioral trajectories, as temporal resolution depends on discrete staged samples rather than real-time tracking. Recently, short-term live confocal and light-sheet imaging combined with optimized ovule in vitro culture has enabled transient monitoring of MMC-specific reporter dynamics, such as pKNU and chromatin remodeling markers, capturing key molecular transitions during MMC differentiation. Nevertheless, extended time-lapse imaging inevitably induces phototoxicity, physiological stress, and growth arrest, preventing uninterrupted recording of the entire specification timeline. Notably, genetic lineage tracing systems, including inducible Cre-Lox recombination and photoconvertible fluorescent markers (Kaede, Dendra2), which have successfully resolved cell ontogeny in vegetative plant tissues, have not yet been adapted to trace MMC origin and clonal relationships. This major gap prevents researchers from definitively answering whether the MMC is predetermined from a fixed L2 precursor pool or selected dynamically from a pluripotent nucellar cell population. Future breakthroughs will depend on integrated technical advances: long-term low-phototoxicity light-sheet imaging, microfluidic ovule cultivation systems, and cell-type-specific inducible lineage-tracing tools. Establishing a continuous molecular and morphological timeline will be essential to disentangle the early sequence of permissive epigenetic changes, hormonal gradient establishment, and lateral inhibitory signaling, and ultimately resolve whether MMC specification is driven by cell-intrinsic developmental programs or extrinsic positional signals from neighboring somatic cells.

Based on these problems and challenges, future research may be advanced in the following directions. Single-cell RNA-seq, single-cell ATAC-seq, single-cell DNA methylation sequencing and other single-cell mul-ti-omics approaches can reconstruct the developmental lineage trees and epigenetic trajectories of MMCs and their adjacent cells, uncovering the spatiotemporal regulatory logic governing cell fate determination. Combining multiple-mutant genetic analysis, synthetic biological reconstitution, and mathematical modeling can elucidate how core regulatory circuits including SPL/NZZ–ARF–PIN1 and ERf–BZR1–WRKY23 coordinately inte-grate developmental and hormonal signals to establish the “one MMC” developmental output. Temporally induci-ble CRISPR/dCas9-based epigenetic editing systems enable targeted manipulation of DNA methylation or histone modifications at specific genomic loci within precise developmental windows, directly verifying the causal effects of epigenetic alterations on MMC fate acquisition. Proximity labeling techniques (e.g., TurboID, APEX2) combined with mass spectrometry can systematically identify secreted proteins, small peptides, and small RNAs produced by MMCs and their neighboring somatic cells, thereby mapping the complete germline–somatic intercellular signaling network. Finally, functional investigation of homologous genes in crops such as rice, maize and tomato will uncover the evolutionary conservation and species-specific divergence of MMC specification mechanisms, providing theo-retical support for improving crop seed setting rate and yield via targeted modification of female germline devel-opment.Megaspore mother cell specification is the initial step in sexual reproduction in flowering plants. Elucidating its mechanisms not only addresses the fundamental question in developmental biology of how cell fate is determined, but also provides an ideal model for understanding establishment of plant germ cell lineages, epigenetic reprogramming, and intercellular communication. Currently, research on MMC specification in *Arabidopsis* has progressed from describing functions of individual genes to a stage of systematic understanding through dynamic integration of multiple pathways and multiple layers. In the future, with continuous development of high-resolution imaging, single-cell multi-omics, and functional validation technologies, we have reason to expect that a complete molecular regulatory map of MMC specification will be drawn in the near future, thereby offering new theoretical tools and strategies for precise regulation of plant reproductive development and crop genetic improvement.

## Data Availability

No new data were created or analyzed in this study. Data sharing is not applicable to this article.
